# From Histology to Multi-Omics: Review of Chordoma Classification and Its Clinical Implications

**DOI:** 10.3390/cells15090750

**Published:** 2026-04-22

**Authors:** Szymon Piotr Baluszek, Paulina Kober, Mateusz Bujko

**Affiliations:** 1 Laboratory of Molecular Oncology, Maria Skłodowska-Curie National Research Institute of Oncology, 02-781 Warsaw, Poland; mateusz.bujko@nio.gov.pl; 2 Laboratory of Molecular Biology, Maria Skłodowska-Curie National Research Institute of Oncology, 02-781 Warsaw, Poland; paulina.kober@nio.gov.pl

**Keywords:** chordoma, molecular classification, DNA methylation, copy number alterations, chromosomal instability, tumor microenvironment, TGF-β, brachyury, *CDKN2A/B*

## Abstract

**Highlights:**

**What are the main findings?**

- A total of 108 studies that enrolled a total of 6349 individuals reveal a variety of chordoma classification methods.
- Across studies, four major interconnecting themes of classification are identifiable: chromosomal instability; SWI/SNF complex dysfunction; stroma–tumor ratio; and immune microenvironment heterogeneity.

**What are the implications of the main findings?**

- Previous works have identified interesting candidates for theranostic classification.
- There is a pressing need for studies that validate these targets in multi-center, prospective studies.

**Abstract:**

Chordoma is a rare malignant neoplasm of the axial skeleton, arising from notochordal remnants. No approved systemic therapies exist, and the 10-year overall survival is below 60%. Accurate molecular and pathological classification is a prerequisite for improved prognostication and the identification of actionable therapeutic targets; however, molecular classification of chordoma remains significantly less advanced than that of other neoplasms. This narrative review synthesizes proposed classification frameworks for chordoma across histological, radiological, surgical, genomic, epigenomic, transcriptomic, and proteomic domains. PubMed and CENTRAL were searched on 1 February 2026 using five queries: ‘chordoma classification’, ‘chordoma DNA sequencing’, ‘chordoma RNA sequencing’, ‘chordoma methylation’, and ‘chordoma copy number’. Original research articles describing more than one patient and reporting a classification or subtyping framework were included; review articles, case reports, and non-English publications were excluded. Sample size and the use of a validation dataset were identified for each study. Results were synthesized qualitatively. A total of 108 studies encompassing 6349 individuals were included. Across six domains, four cross-cutting themes with prognostic and potential theranostic value emerged: copy number alterations, particularly *CDKN2A/B* loss; SWI/SNF complex dysfunction; stroma–tumor ratio; and immune microenvironment heterogeneity.

## 1. Introduction

Chordoma is a rare primary malignant neoplasm of the axial skeleton arising from remnants of the embryological notochord, with a reported annual incidence of approximately 0.088 per 100,000 persons in the United States. From 2004 to 2014, a total of 3670 chordomas were diagnosed in the US, with the most common location being cranial (38.7%), followed by sacral (34.3%) and spinal (27.0%) [1]. The incidence increases with age, with a clear male predominance [1]. On average, patients are diagnosed in their late fifties, and the incidence varies between countries and presumably between races [2]. The five-, ten-, and fifteen-year overall survival probabilities following chordoma diagnosis are approximately 0.74, 0.58, and 0.48, respectively, with no significant change in the hazard of death observed over the 2003–2019 period [3].

The clinical management of chordoma is challenging and centers primarily on surgical resection followed by high-dose radiotherapy. Gross total resection has an established relationship with progression-free and overall survival; however, the tumor’s anatomical location frequently interferes with attempts at complete resection, and no consensus currently exists on the most effective adjuvant radiotherapy modality [4]. Chordoma is unresponsive to available chemotherapies and to doses of radiation that can be safely delivered with conventional radiotherapy, which substantially limits treatment options [5]. The advent of particle therapy—in particular proton and carbon ion irradiation—has meaningfully expanded the therapeutic armamentarium, as these modalities permit dose escalation to levels exceeding 70 Gy while sparing adjacent critical structures [6]. Nevertheless, even with multimodal therapy, local recurrence remains the most common failure pattern, translating to adverse overall survival [7]. For patients who fail surgery and radiotherapy, there remains an urgent unmet need for new therapeutic options.

In this study, we present a review of the evolving molecular and pathological classification of chordoma, examining the histological, radiomic, clinical, genomic, epigenomic, transcriptomic, and proteomic frameworks that have been proposed to stratify this disease into clinically and biologically meaningful subtypes. Accurate classification is a prerequisite to improved prognostication, the identification of actionable therapeutic targets, and rational clinical trial design. Over the years, numerous prognostic, diagnostic, and predictive markers were described. Despite this, the contribution of molecular features to chordoma classification remains significantly less advanced than in the official classification systems for other neoplasms. Here, we provide a narrative review with systematic search elements and attempt to synthesize various classifiers and indicate gaps in knowledge that should be addressed in future research.

## 2. Materials and Methods

### 2.1. Search

A comprehensive search of English-language literature was conducted on 1 February 2026. PubMed^®^ and CENTRAL were accessed and searched for the following phrases: ‘chordoma classification’, ‘chordoma DNA sequencing’, ‘chordoma RNA sequencing’, ‘chordoma methylation’, and ‘chordoma copy number’. No additional restrictions or Boolean logic were set.

### 2.2. Eligibility Criteria and Study Selection

Studies were screened, and any doubts among the reviewers were resolved through discussion. The selection process had two steps. First, the abstracts of the identified articles were accessed. Review articles, case reports, articles pertaining to other pathologies and articles in languages other than English were excluded. Second, full-length articles were accessed and searched for relevance to chordoma classification. If a study proposed a biomarker or classification system that stratified a chordoma patient population or was useful for distinguishing it from other similar pathologies, it was included. This approach is summarized in Table 1.

### 2.3. Data Extraction

Data were summarized in a table, with predefined columns: area of interest, year of publication, technology, number of patients, and presence of a validation cohort. The data were organized to enable meaningful comparisons and synthesis of findings. These data are available in Supplementary Table S1.

### 2.4. Assessment of Risk of Bias of Individual Studies

Given that the studies were conducted across a wide variety of settings and time periods, and that entities diagnosed as chordoma may differ between studies (see Section 3 and Section 3.1), the risk of bias for each included study could result from factors beyond the researchers’ control. Nonetheless, study size and attempts at replication in an independent chordoma cohort were noted. These two identifiable factors, related to risk in clustering studies, are included in Supplementary Table S1.

### 2.5. Results Presentation

Given the nature and variety of the studies included, the most productive approach was a qualitative, narrative review. After concluding Section 3, the text was screened for themes that spanned more than one section. Such themes were selected for further discussion. R 4.5.2 along with ggplot2, was utilized to summarize and visualize the results. Both the search results and the visualization program are available as Supplementary Materials. Generative AI was utilized (Sonnet 4.6) for grammar and language checks.

## 3. Results

**Figure 1:**
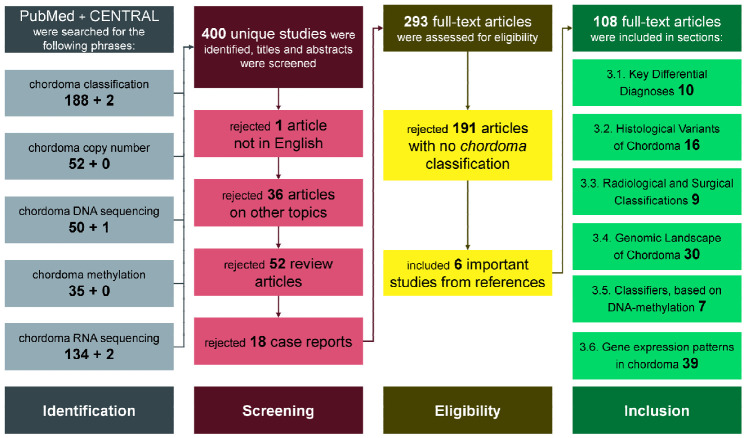
Flowchart of the search process. The total number of articles included in the review was 108; three articles [8,9,10] were selected for two sections (thus a cumulative number of 111 after adding the numbers in each section).

In total, the queries yielded 464 articles, and after removing duplicates, 400 unique articles remained. Subsequently, 293 passed the initial abstract screening, and after full-text review, 102 were deemed relevant to chordoma classification. In this process, an additional six studies were included from the references of the selected articles. See Figure 1 for more granular information. Nineteen studies included at least 100 patients, 39 included at least 50 patients, and 67 at least 25 patients. Fourteen studies utilized a validation dataset.

**Figure 2:**
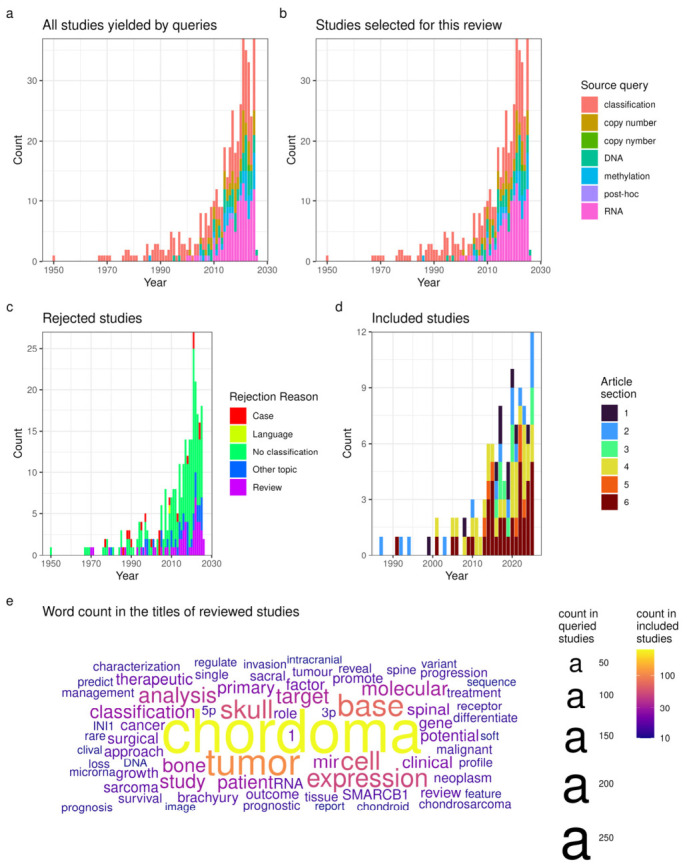
Summary of the review: (**a**) query results for all studies, split by year; (**b**) query results of studies included in the article, split by year; (**c**) causes for article rejection, split by year; (**d**) article sections where the included studies were cited, split by year; (**e**) word cloud from titles of all the articles queried (>10 occurrences per word). The size relates to the number of times a word appeared in all titles, while the color scale relates to the number of times the same word appears in the titles of articles included in the review. The terms “1” refer to chromosome numbers; “5p” and “3p” come from both chromosome arm names and mi-RNA terminology.

Overall, most of the articles (349/406 = 400 identified in search + 6 identified during the review) were published after 2000, with a trend towards increased publications most pronounced for the ‘chordoma RNA sequencing’ query (see Figure 2a,b). The most common cause for study rejection was the lack of an included classification of chordoma cases (see Figure 2c). Of the 108 included studies, 103 were published after 2000, with the visible growth mainly in articles reviewed in Section 3.6, dealing with gene expression patterns in chordoma (see Figure 2d). Recurring words (>10 occurrences) in the titles of the queried articles are depicted in Figure 2e. The number of times these words appeared in the titles of included articles is color-coded, showing consistency of coverage. Note words related to experimental methods (e.g., DNA, RNA, mir), frequent anatomical locations of chordoma (e.g., sacral, clival, spine), and molecular findings (e.g., brachyury, INI1, and 1 from ‘chromosome 1’).

### 3.1. Key Differential Diagnoses

Before establishing classification patterns within a neoplasm entity, it is crucial to define lesions representing such a neoplasm. According to the WHO Classification of Tumors of Soft Tissue and Bone, the key distinguishing features of chordoma are a lobular growth pattern, cells embedded in a rich basophilic mucoid or myxoid matrix, and physaliphorous cells (large, polygonal cells with abundant, pale, *bubbly* or vacuolated cytoplasm) [11].

The first distinction is between chordoma and chondrosarcoma, as they represent distinct pathological entities with different embryonic origins and anatomical predilections. Chordomas arise from the remnants of the primitive notochord, whereas chondrosarcomas originate from the cartilaginous matrix of the skull base synchondroses [12]. An important diagnostic differentiator is anatomical location, as chordomas are predominantly midline structures involving the clivus, while chondrosarcomas are typically situated off-midline, most frequently at the petro-occipital fissure [13]. Furthermore, while chondrosarcomas are often classified as histological Grade I or II and exhibit a relatively indolent clinical course, chordomas are characterized by a more locally invasive and aggressive growth pattern [14]. Ultimately, these biological differences translate into divergent survival outcomes; chondrosarcomas are associated with significantly better long-term prognoses, with five-year survival rates often reaching 80% to 90%, whereas chordomas generally demonstrate lower five-year survival rates of approximately 65% [12].

Radiographic characteristics and clinical outcomes further distinguish these two malignancies. Chondrosarcomas frequently exhibit characteristic intratumoral calcifications on computed tomography, a feature less common in chordomas [14]. Quantitative analysis using diffusion-weighted magnetic resonance imaging indicates that chondrosarcomas typically have higher apparent diffusion coefficient values than chordomas, reflecting the former’s lower cellularity and higher water content within the cartilaginous matrix [15]. Histopathologically, the two entities differ significantly. Chordomas stain positive for brachyury and cytokeratins (i.e., cytokeratin 8, 18, and 19) and lack the chondroid-like cells embedded in the lacunae typical of chondrosarcoma [11]. Chondrosarcomas tend to be affected by *IDH1/2* mutations more often than chordomas [16], although these mutations in chordomas have also been reported [10].

Parachordoma is an exceedingly rare soft tissue tumor (~40 reported cases) [17] that mimics axial chordoma but arises in extra-axial locations. Historically recognized as *chordoma periphericum* or *chordoid sarcoma*, the neoplasm typically presents as a slow-growing, painless mass, often involving deep fascial tissues. Microscopic examination reveals a lobulated architecture consisting of nests and cords of epithelial-like rounded cells with abundant, often vacuolated cytoplasm, occasionally forming structures that resemble the embryonal notochord. The extracellular matrix generally appears myxoid, chondroid, or hyaline in consistency. Immunohistochemical analysis is essential for definitive diagnosis, as the neoplastic cells demonstrate a bimodal differentiation, indicated by strong expression of vimentin alongside focal expression of epithelial markers such as cytokeratin (peptides 8 and 19) and EMA, as well as variable expression of S-100 protein. This epithelium-specific immunoreactivity pattern is critical for differentiating parachordoma from extraskeletal myxoid chondrosarcoma, which lacks such markers [18]. Parachordomas do not express brachyury (encoded by the *TBXT* gene), in contrast to extra-axial chordomas [19]. Current literature suggests that parachordoma is a mesenchymal neoplasm with a generally good prognosis following surgical excision [17].

Another clinically important entity to be distinguished from chordoma is benign notochordal cell tumor (BNCT), also known as *ecchordosis physaliphora*. It is a congenital, histologically benign hamartomatous remnant of the embryological notochord, typically located along the craniospinal axis, most frequently at the level of the clivus [20,21]. It is reported in up to 2% of autopsies [20]. Radiologically, the lesion is characterized as a well-circumscribed, T2-hyperintense, non-enhancing mass, often distinguished from other retroclival lesions by a bony stalk or pedicle projecting from the clivus [21,22]. Although historically regarded as an asymptomatic incidental finding, BNCT can precipitate life-threatening complications, including spontaneous cerebrospinal fluid rhinorrhea and recurrent bacterial meningitis associated with midline clival defects [21]. Histopathologically, BNCT comprises physaliphorous cells with vacuolated cytoplasm and a very low Ki-67 proliferation index of less than 1%; yet, significant radiological and histological overlap with low-grade chordomas suggests a spectrum of disease rather than a strict benign–malignant dichotomy [22,23]. Therefore, the creation of a separate entity, *atypical notochordal cell tumor,* to encompass the in-between lesions has been proposed [24].

### 3.2. Histological Variants of Chordoma

According to the WHO Classification of Tumors of Soft Tissue and Bone, the vast majority of chordoma cases represent conventional chordoma, which is characterized by a lobular growth pattern, along with cells embedded in a mucoid/myxoid matrix, and physaliphorous cells. However, there are two rarer forms of chordoma: dedifferentiated chordoma (DC) and poorly differentiated chordoma (PDC) [11]. Further subtypes within the realm of conventional chordoma can be described. In a recent study evaluating the ultrastructural features of clival chordomas, these tumors were stratified into cell-dense (CDT) and matrix-rich (MRT) subtypes using electron microscopy. Their analysis revealed that the CDT variant is associated with a significantly higher Ki-67 proliferation index, an increased risk of early recurrence, and higher mortality rates compared to MRT chordomas [25].

There exists some controversy regarding chondroid chordoma (ChoC). It is a morphological variant of conventional chordoma characterized by a biphasic growth pattern containing both conventional chordoma elements and areas of cartilaginous differentiation [11,26]. While historically debated and occasionally misclassified as low-grade chondrosarcoma due to morphological similarities [27], its classification as a true chordoma was definitively established through immunohistochemical profiling. Unlike low-grade chondrosarcomas, which are mesenchymal tumors lacking epithelial markers, the cells within both the conventional and cartilaginous components of chondroid chordomas consistently express brachyury and epithelial markers such as cytokeratin, epithelial membrane antigen, and carcinoembryonic antigen [28]. Consequently, immunohistochemistry serves as the critical diagnostic tool to differentiate true chondroid chordomas from chondrosarcomas. Accurate clinicopathological classification is essential because ChoC is generally associated with a more favorable prognosis and better overall survival compared to classic chordoma [28]. In the newest WHO Classification, ChoC is described as a variant of conventional chordoma [11].

Dedifferentiated chordoma is a rare and highly aggressive malignancy, comprising less than one percent of all chordomas [29]. It is histologically defined by a biphasic appearance in which a typical conventional chordoma is directly juxtaposed with a high-grade sarcomatous component [29]. During the process of dedifferentiation, the sarcomatous regions characteristically lose the expression of key diagnostic lineage markers, notably brachyury and cytokeratin, which remain conserved in the adjacent conventional tumor cells [29]. Clinically, the onset of dedifferentiation portends an extremely poor prognosis, characterized by rapid disease progression and a median overall survival of approximately twenty months [29]. The molecular landscape of DC is distinct from its conventional counterpart. The transition is frequently associated with recurrent mutations in *TP53*, which are found in a significant subset of these tumors and serves as a major driver of the aggressive phenotype with prognostic value [30]. *TP53* loss-of-function can be limited to the dedifferentiated parts of the tumor [29]. Moreover, it has been shown that even in the cohort of conventional chordomas, *hTERT* expression, linked to *TP53* dysfunction, is a prognostic factor [31]. Furthermore, dedifferentiation may arise de novo or secondary to radiation therapy [32]. This behavior in skull base chordomas (SBCs) was characterized by a unique epigenetic shift and specific somatic alterations, including *PIK3CA* mutations [32]. Additionally, a distinct Polycomb-type dedifferentiation pathway has been identified in a specific subset of skull base dedifferentiated chordomas [33]. This variant is often driven by homozygous deletions of *EED*, leading to a targeted loss of H3K27 trimethylation exclusively within the malignant sarcoma-like component, often resulting in a histology that mimics malignant peripheral nerve sheath tumors [33].

Poorly differentiated chordoma represents another rare and aggressive subtype of chordoma, frequently located in the skull base or cervical spine and occurring often in the pediatric population [34,35,36]. In the adult population, extra-axial PDC has been reported [37,38]. Unlike conventional chordoma, poorly differentiated chordomas exhibit an atypical, high-grade morphology consisting of cellular proliferations of epithelioid to rhabdoid cells with prominent nucleoli and vesicular nuclei, often lacking the hallmark bubble-like vacuolization [39]. A defining molecular feature of this entity is the recurrent loss of *SMARCB1* (INI1) expression, a core subunit of the SWI/SNF chromatin remodeling complex, which serves as a critical diagnostic marker [39,40]. Despite the loss of *SMARCB1*, these tumors maintain strong nuclear expression of brachyury. This expression profile reliably distinguishes poorly differentiated chordomas from other INI1-deficient tumors, i.e., atypical teratoid/rhabdoid tumors, which are consistently brachyury-negative [34]. The clinical progression of poorly differentiated chordoma is notably more rapid than that of conventional chordoma, characterized by a high propensity for early local recurrence, metastatic spread, and increased mortality [34]. The identification of *SMARCB1* loss has not only refined the diagnostic criteria for these tumors but has also provided a mechanistic rationale for targeted epigenetic therapies. Specifically, *SMARCB1* deficiency leads to unopposed oncogenic activity of Enhancer of Zeste Homolog 2 (EZH2), prompting the clinical investigation of EZH2 inhibitors. While a sustained antitumor response to tazemetostat has been reported in a single case of metastatic PDC harboring homozygous *SMARCB1* loss, these preliminary findings require validation in larger, prospective clinical trials to determine the broader therapeutic efficacy of this approach [41].

### 3.3. Radiological and Surgical Classifications

For diagnostic purposes, the majority of patients with chordoma undergo MRI. Capitalizing on this, radiological classification of SBCs has been proposed. It utilizes normalized signal intensity ratios derived from T1-weighted FLAIR, T2-weighted, and contrast-enhanced MRI sequences compared against the pons [42,43]. The ratio of tumor-to-pons signal intensity on T2 sequences (RT2) and contrast-enhanced T1 sequences can serve as a primary prognostic indicator, identifying high-grade tumors, which are associated with increased tumor blood supply, greater intraoperative blood loss, and progression risk [42]. These imaging characteristics correlate with the underlying electron microscopic ultrastructure, where MRT chordomas typically exhibit higher RT2 values and more favorable long-term outcomes compared to CDT chordomas [43]. Furthermore, advanced radiomic analysis of T2-weighted imaging features has demonstrated clinical utility in constructing predictive signatures for four-year recurrence probability, with high-dimensional 3D features achieving accuracy rates of approximately 85% in distinguishing high-risk tumor profiles before surgical intervention [44].

Radiogenomics and radiomics provide emerging non-invasive methodologies for the diagnostic and prognostic evaluation of chordomas by correlating macroscopic imaging characteristics with microscopic, molecular, and epigenetic profiles [45,46,47]. In sacral lesions, 3D computed tomography radiomics facilitates the accurate differentiation of chordomas from giant cell tumors [45]. For SBCs, MRI radiomics can predict both clinical outcomes and some DNA copy number alterations (CNA), i.e., losses of chr1p and chr9 [46]. Furthermore, radiogenomic profiling has linked a specific prognostic DNA methylation profile to a 14-feature MRI signature in SBCs [47].

Surgical classification schemes of SBCs are primarily utilized to guide operative strategy and optimize resection margins, which correlate strongly with long-term progression-free survival [48]. A recent retrospective analysis revealed that the sphenoclival region is the most affected and that the pattern of bone invasion is predominantly endophytic [48]. More specific staging systems categorize clival chordomas into distinct types (I—dorsal clivus, II—ventral clivus, III—inferior third of the clivus, and IV—paramedian) [49]. These classification frameworks are critical for determining the suitability of minimally invasive endoscopic endonasal approaches versus traditional open cranial base approaches [50]. By applying these classification schemes preoperatively, surgeons can better predict the feasibility of achieving marginal or gross total resection, identify highly probable sites of residual tumor tissue such as the cavernous sinus, petrous apex, or parapharyngeal space, and ultimately minimize surgical morbidity while maximizing therapeutic efficacy [48,49,50].

### 3.4. Genomic Landscape of Chordoma

Although the majority of chordoma cases are sporadic, familial susceptibility has been described [51,52,53]. Our current understanding of genetic susceptibility to chordoma implicates a complex, polygenic landscape involving both rare and common germline alterations. The foundational driver of chordoma risk in familial predisposition cases involves germline duplications and specific sequence variants in the *TBXT* gene [51]. A high frequency of the common *TBXT* susceptibility variant rs2305089 was additionally confirmed in 97.8% of patients in a French cohort of SBCs and spinal chordomas, significantly elevated compared to the general population [54]. Beyond *TBXT*-related mechanisms, rare germline variants in homologous recombination genes, notably *BRCA2* and *PALB2*, have been identified in familial and sporadic cohorts, highlighting DNA repair deficiency as a pathogenic factor and potential therapeutic vulnerability [52]. Results from *broader* DNA sequencing indicate additional rare loss-of-function and missense germline variants distributed across diverse oncogenic and developmental signaling networks, including the SWI/SNF, PI3K/AKT/mTOR, and Sonic Hedgehog pathways [53]. Furthermore, common single-nucleotide polymorphisms, e.g., in the *LGALS3* gene, contribute to this multifactorial susceptibility by elevating the risk of SBC and predicting reduced progression-free survival following treatment [55].

Chordoma is characterized by a low overall somatic mutation burden relative to most other cancer types [10,30,56]. Whole-genome and whole-exome sequencing studies have consistently identified a limited repertoire of recurrently mutated driver genes. Alterations in SWI/SNF chromatin remodeling complex genes are among the most frequent and best-characterized events. *PBRM1* mutations or structural variants have been reported across skull base, spinal, and sacral cohorts, and co-occurring alterations in *SETD2* and *ARID1A* further implicate defective chromatin remodeling as a central oncogenic mechanism [10,54,56,57,58]. Activating mutations in PI3K pathway genes, predominantly *PIK3CA*, occur in approximately 10–16% of tumors across multiple cohorts [54,56,59]. While these alterations are considered potential therapeutic targets, their clinical utility as predictive biomarkers for PI3K/AKT/mTOR inhibitor sensitivity in chordoma remains to be established in a clinical setting. *LYST*, encoding a lysosomal trafficking regulator, was identified as a candidate novel chordoma driver gene through recurrent truncating mutations in approximately 10% of sporadic cases. A speculative mechanistic link to the lysosomal biology of notochordal cells has been proposed, though this requires further functional validation [56]. Early targeted panel sequencing identified mutations in *KDR* and *KIT* in small cohorts, though these remain infrequent and their functional driver significance is uncertain [60].

It was observed that half of chordoma cases lack identifiable driver mutations [10,56]. However, CNAs are frequent, and chromosomal instability (CIN) has been identified as a prognostic marker [8,9,59]. The changes are consistent between primary and recurrent or metastatic samples, suggesting a foundational role in tumor development [10]. More specifically, homozygous deletion of the *CDKN2A/B* (on chr9p) locus is recurrent and was more commonly observed in spinal and recurrent tumors in two independent cohorts [10,54,61]. There is some controversy surrounding the prognostic significance of these alterations: Horbinski et al. observed worse overall survival in chordomas with 9p loss of heterozygosity, while homozygous 9p21 deletion only trended toward significance without reaching it [62]. Meanwhile, Passeri et al. observed that the homozygous loss of *CDKN2A/B* confers worse prognosis and is mutually exclusive with mutations affecting the SWI/SNF complex in a mixed sacral, spinal and SBC cohort [54]. However, in an SBC population, Bai et al. found that *CDKN2A/B* status was not independently associated with chordoma specific survival, but it was associated with worse progression-free survival [10]. Since *CDKN2A/B* genes encode p16 (INK4A) and p15 (INK4B), negative regulators of cyclin-dependent kinases 4 and 6 (CDK4/6), the high frequency of chromosome 9p loss has sparked interest in targeting cell cycle regulation in chordoma. A non-randomized phase II trial PMO-1601 showed modest antitumor activity of CDK4/6 inhibitor palbociclib in *CDKN2A/B*-deleted tumors; further investigation is necessary to define the subset of patients most likely to derive a durable clinical benefit [63,64].

Beyond that, recurrent chromosomal arm or whole-chromosome losses include deletions of 1p, 3, 4, 5, 9p, 9q, 10, 13q, 14q, 18, and 22q, while gains of 1q and 7 are also observed [10,59,65,66,67,68,69,70,71,72,73]. Deletion of 22q, which harbors *SMARCB1*, was associated with worse disease-specific and recurrence-free survival in SBC, and the combination of *PBRM1* point mutations with 22q deletion further strengthened this prognostic association [10]. However, loss of INI1 staining in conventional SBC was not associated with worse prognosis in another cohort [74]. Nonetheless, homozygous loss of *SMARCB1* is linked with PDC [34,35,36,37,38,39,40,41], and it is not yet clear if heterozygous loss is a transition state towards this pathology. Somatic deletions of 14q and 18p were associated with persistent or recurrent disease in a North American cohort spanning all anatomical sites [59]. Genomic diversity by tumor site is well documented: sacral chordomas harbored driver gene mutations, *TBXT* amplifications, and deletions of 5p, 5q, and 9p more frequently than clival tumors [10,59,75]. We have recently proposed a four-group classification scheme for CNA in chordoma [64]:

- C1 for chromosomally stable tumors;
- C9 for tumors with predominant chromosome losses, e.g., chr9p; these tumors tend to also have a 22q loss;
- C7 for tumors with predominant chromosome gains, especially chr7;
- C2 for tumors with both gains and losses (i.e., both chr9p loss and chr7 gain); gain of chr2 seems to be characteristic for that cluster. Notably, gain of chr2 was independently associated with a higher recurrence rate in SBC [76].

### 3.5. Classifiers, Based on DNA Methylation

Accumulating evidence indicates that aberrant DNA methylation is a significant epigenetic feature of chordoma pathobiology. Rinner et al. provided early evidence of this by identifying 20 differentially methylated genes compared to peripheral blood—including hypermethylation of the tumor suppressor genes *RASSF1*, *HIC1*, and *KL*—with a multigene methylation-based classifier able to distinguish chordoma from healthy blood DNA [77]. Marucci et al. demonstrated that *MGMT* promoter methylation is present in a significant proportion of recurring clival chordomas, while remaining consistently unmethylated in non-recurring cases [78]. Since the efficiency of temozolomide treatment has been previously shown as related to *MGMT* promoter methylation in other cancer types [79], this observation raised the possibility that temozolomide may have a role as adjuvant therapy in recurrent chordoma cases; however, no prospective clinical trial has been completed yet. Alholle et al. performed genome-wide DNA methylation profiling of 26 chordomas and normal nucleus pulposus samples, identifying 8819 significantly differentially methylated loci; functional analysis indicated that genes affected by cancer-specific methylation changes were involved in networks including cancer disease, nervous system development and function, and cellular proliferation [80].

DNA methylation profiling has emerged as a reliable molecular tool for the classification and prognostic stratification of chordomas. Across independent cohorts, unsupervised clustering of genome-wide methylation data consistently identifies two distinct epigenetic subtypes [8,81,82]. One subtype, variously termed *immune-infiltrated* or *Chordoma I*, is characterized by relative global hypermethylation, higher immune cell infiltration, including cytotoxic T lymphocytes, B cells, neutrophils, and macrophages, and lower tumor purity [8,81]. The other subtype—*cellular* or *Chordoma C*—displays global hypomethylation with CpG island hypermethylation, higher tumor purity, and greater chromosomal instability [8,81]. *CDKN2A/B* locus deletions are associated with the subtypes, though the specific cluster association differs between studies: Huo et al. reported *RB1* and *CDKN2A/B* deletions predominantly in their immune-infiltrated *Cluster 1,* while Baluszek et al. found 9p deletions in nine of ten *Chordoma C* tumors [82]. The clusters were equivalent with each other; in the latter publication, samples from Huo et al. were reanalyzed, and *Cluster 1* grouped with *Chordoma C* [8]. They also shared global methylation patterns and estimates of cytotoxic T-cell infiltration [8]. Both Huo et al. and Zuccato et al. reported worse prognosis in the immune-infiltrated subtype, while we have not observed such effect in our cohort [8,81,82].

Beyond subtype classification, methylation profiling has shown translational promise in two additional directions. Plasma cell-free DNA methylomes, obtained via cfMeDIP-seq, were shown to distinguish chordomas from clinical mimics such as meningiomas and spinal metastases, and leave-one-out models correctly assigned all twelve paired tumors to their tissue-based methylation subtype using plasma-derived signals alone, demonstrating the feasibility of non-invasive prognostication [81]. A three-CpG-site prognostic risk score, incorporating loci mapped to *BATF*, *ACTR3C*, and *FGFBP2*, achieved AUC values of 0.775, 0.795, and 0.904 for three-, five-, and ten-year survival, respectively [82]. In the broader sarcoma context, the DKFZ Sarcoma Classifier—a random forest tool trained on 1077 reference samples across 54 bone and soft tissue subtypes—correctly classified chordomas in 85% of cases (75/88 samples) in an independent external validation cohort of 986 bone and soft tissue tumors, making chordoma one of the entities most reliably identified by this approach [83]. Further prospective investigation is needed to properly assess the utility of DNA methylation arrays in routine clinical practice.

### 3.6. Gene Expression Patterns in Chordoma

Chordomas universally express simple epithelium cytokeratins 8, 18, and 19 alongside vimentin, EMA, and S100, while lacking desmin and squamous-type cytokeratins, reflecting their notochordal origin [84,85,86,87]. Pediatric cases more frequently exhibit p53 expression, INI1 loss, and elevated MIB-1 labeling indices, features associated with more aggressive clinical behavior [85]. Brachyury expression levels correlate with shorter progression-free survival and with upregulation of PI3K/Akt pathway gene expression in SBCs [88]. Somatic copy number gain of the *TBXT* (brachyury) gene locus has been identified in a subset of cases [89]. EGFR, c-Met, and HER2/neu are detectable by immunohistochemistry across chordomas, with most tumors showing strong expression of both c-Met and EGFR [90,91]. Overexpression of *MET* in chordoma is not caused by gene fusions, as commonly observed in sarcomas [92]. Tamborini et al. observed that *PDGFRB* is highly expressed and phosphorylated in sacral and lumbar chordoma, while *PDGFRA* and *KIT* have lower expression levels but are nonetheless activated by phosphorylation. Since no gain-of-function mutations nor gene amplification were identified in that study, an autocrine or paracrine ligand-driven activation loop was proposed as the underlying cause [93]. PDGFR-β expression additionally correlates with invasive behavior via the mTOR signaling pathway and predicts shorter progression-free and overall survival in patients with clival chordoma who undergo non-total resection [94]. Finally, high *PALB2* expression independently predicts shorter progression-free survival in SBC and promotes proliferation, migration, and invasion in vitro, implicating dysregulation of DNA homologous recombination repair as a contributor to chordoma pathobiology [95].

Gene expression profiling has revealed significant molecular heterogeneity in chordoma. RNA sequencing of SBCs identified two immune subtypes differing in the degree of macrophage and T-cell infiltration, with higher *CD68* and *CD163* expression correlating with shorter progression-free and overall survival [96]. Similar transcriptomic analysis of SBCs distinguished two molecular subtypes: *CC1* tumors were associated with somatic mutations in chromatin remodeling genes such as *PBRM1* and *SETD2*, while *CC2* tumors exhibited upregulation of Sonic Hedgehog pathway genes, with expression of markers such as *PTCH1* showing prognostic significance [97]. Gene expression patterns are also linked to the stromal component of SBCs; these tumors can be classified into stroma-rich and stroma-poor cases (similar to CDT and MRT types described earlier). Stroma-rich chordomas are more often enhanced in MRI studies, have higher expression of genes associated with tumor metastasis and progression, and show a tendency towards poorer prognosis [98]. There exists some inconsistency regarding the prognostic value as Bai et al. report MRT to have longer expected survival [43].

Recent single-cell RNA sequencing (scRNA-seq) studies have corroborated these findings while substantially advancing the understanding of chordoma biology, heterogeneity, and therapeutic targeting. First, Duan et al. identified a varied tumor microenvironment in chordomas from the skull base, mobile spine, and sacrum, with numerous infiltrating M2-like macrophages and T/NK lymphocytes and marked TGF-β signaling, mainly between macrophages and fibroblasts [99]. These findings were confirmed in SBCs by Zhang et al., who additionally identified a stem cell-like malignant cell subpopulation—marked by *CTSL* expression—associated with radioresistance through a telomere-end packaging mechanism. The same study described a partial epithelial–mesenchymal transition (p-EMT) program, driven by TGF-β, that localized to the invasive tumor edge and predicted worse progression-free and overall survival [100]. Extending this research, scRNA-seq analysis on 14 SBC samples identified VEGFR and TGF-β as co-dominant therapeutic targets. Subsequently engineered dual VEGFR/TGF-β CAR-T cells demonstrated superior and sustained cytotoxicity over VEGFR-only CAR-T cells against chordoma cell lines and patient-derived organoids [101]. Complementarily, in SBCs, a basement membrane-related gene signature comprising five genes—*ITGB3*, *SMOC1*, *UNC5B*, *COL14A1*, and *COL13A1*—was independently associated with recurrence-free survival, immune cell infiltration patterns favoring immunosuppression and differential drug sensitivity, with *ITGB3* knockdown functionally impairing chordoma cell proliferation and migration via the PI3K-Akt pathway, confirming the relevance of TGF-β signaling [102].

Bridging the stroma and immune microenvironment research, multi-omics profiling, including spatial transcriptomics and multiplexed immunofluorescence across large patient cohorts, demonstrated that a specific inflammatory cancer-associated fibroblast subset, while spatially distant from tumor cells, correlates with malignant phenotypes and adverse outcomes [103]. Furthermore, Huo et al. applied scRNA-seq alongside T- and B-cell receptor sequencing to compare primary and recurrent SBCs, finding that recurrent tumors displayed reduced antigen-presenting cell activity, fewer plasma cells, and contracted BCR clonotype diversity. Fibronectin 1 (*FN1*) was upregulated in recurrent disease and secreted by tumor cells, macrophages, and lymphocytes. It promoted chordoma invasion and proliferation in vitro and in vivo. The study further identified exhaustion of CD8^+^GZMK^+^ T cells and M2-like macrophage polarization as key immunosuppressive mechanisms associated with recurrence [104]. Additionally, chordoma progression is driven by metabolic–immune crosstalk via the BACH1/ANGPTL4/SDC4 signaling pathway, wherein cholesterol-metabolic tumor-associated macrophages induce stemness and cholesterol accumulation in tumor budding-like cell subpopulations [105]. These studies build on earlier immune research in the field, which showed that *PD-L1* expression is predominantly localized to tumor-infiltrating macrophages and lymphocytes rather than chordoma cells in vivo, although expression remains inducible in chordoma cell lines [106]. Furthermore, the anti-apoptotic protein survivin (*BIRC5*) maintains cell cycle progression in sacral chordomas, and its pharmacological inhibition results in G2/M phase arrest, increased polyploidy, and apoptosis [107].

Several other molecular markers have been investigated individually for their prognostic and therapeutic relevance in chordoma through gene expression studies. Two independent studies implicated developmental gene expression in worse prognosis in SBCs; they both built gene signatures associated with fetal development and found them to be prognostic in chordoma. However, the composition of the signatures differed [108,109]. *CDK12* expression, assessed by tissue microarray in 56 spinal chordoma specimens, was significantly elevated in recurrent and metastatic disease and independently predicted shorter overall and progression-free survival; siRNA-mediated CDK12 knockdown attenuated chordoma cell growth in vitro [110]. Similarly, in spinal chordoma, high *CDK9* expression correlated with recurrence and poor clinical outcomes, and selective pharmacological inhibition with LDC000067 reduced cell proliferation, induced apoptosis, and suppressed colony and spheroid formation [111]. In SBCs, *TGFB1* mRNA levels were significantly higher in hard-type versus soft-type tumors and in female versus male patients across a cohort of 57 cases, with elevated expression associating with tumor progression [112]. The expression of two genes encoding RNA-binding proteins that regulate post-transcriptional gene expression, *SAM68* and *IMP3,* was examined in sacral chordoma, and their expression was associated with shorter progression-free survival [113,114].

Noncoding RNAs (ncRNAs) have also emerged as significant regulators of chordoma pathobiology. Integrated miRNA-mRNA profiling comparing chordoma tissues with fetal notochord identified 33 significantly dysregulated miRNAs and 2791 differentially expressed mRNAs, whose expression was associated with these miRNAs. Pathway analysis implicated MAPK signaling as the most overrepresented oncogenic pathway [115]. A complementary experiment in sacral chordoma and nucleus pulposus samples identified even broader miRNA-mRNA regulatory network associated with tumorigenesis and immune modulation. Notably, it revealed a previously uncharacterized miRNA/mRNA axis involved in stemness regulation, with molecular components proposed as candidate therapeutic targets [116]. The role of individual noncoding RNAs was also described; miR-1—previously shown to suppress chordoma cell growth by targeting *MET*—has been demonstrated to carry prognostic significance in chordoma patients, supporting a functional role for this miRNA in disease progression [117].

Proteomic analyses of chordoma have progressively advanced from early differential profiling to large-scale multi-omics integration, collectively revealing molecular heterogeneity that carries both prognostic and therapeutic implications. An iTRAQ-based quantitative proteomic study of clival chordoma classified tumors by degree of bone invasion into endophytic and exophytic subtypes. The results indicated 250 differentially expressed proteins and implicated TGFβ1 downregulation and reduced PTEN expression in aggressive bone infiltration, with the PI3K/AKT/mTOR pathway proposed as a mediator [118]. Next, the results of two tandem mass tag-based proteomic studies were published. The first identified ASNS as a significantly upregulated protein in rapidly recurring SBC and subsequently validated it by immunohistochemistry in a 187-patient tissue microarray SBC cohort, where high ASNS expression predicted shorter recurrence-free survival [119]. The second study included 102 chordoma patient samples from various anatomical locations and described three molecular subtypes—bone microenvironment-dominant, mesenchymal-derived, and mesenchymal-to-epithelial transition-mediated—with distinct biological features encompassing osteoclastogenesis and immunogenicity, oxidative phosphorylation, and receptor tyrosine kinase activation, respectively. This study matched these subtypes to theranostic therapies with denosumab, S-Gboxin, and anlotinib and validated them in patient-derived xenograft models [120]. The largest and most comprehensive multi-omics study to date integrated whole-exome sequencing, RNA sequencing, proteomics, and phosphoproteomics across 187 SBCs. It pointed out CIN as a prognostic predictor, with CIN-high status correlating with elevated DNA replication stress, E2F3 transcriptional activity, and CDK4-mediated RB1 phosphorylation. Chromosome 1q gain was further associated with mitochondrial pathway upregulation and poor outcomes, while immune subtyping revealed a cold immune subtype linked to chr9p/chr10q loss. Moreover, proteomics-based classification identified invasive subtypes with potential therapeutic vulnerabilities, including *RPRD1B* as a target in radiotherapy-resistant disease [9].

## 4. Discussion

This review synthesizes evidence from 108 studies encompassing 6349 individuals to provide a comprehensive, multi-domain classification framework for chordoma across pathological, clinical, and molecular dimensions. First, we delineated the principal differential diagnostic entities and the immunohistochemical and radiological tools used to distinguish them. Second, we described the established histological variants of chordoma, each carrying distinct molecular features and prognostic implications. Third, we reviewed radiological and surgical classification frameworks, including emerging radiomic approaches that link imaging phenotypes to underlying molecular and epigenetic profiles. Fourth, we characterized the genomic landscape of chordoma, highlighting its low somatic mutation burden, the predominance of chromatin remodeling gene alterations, and recurrent CNAs. Next, we reviewed DNA methylation-based classifiers that demonstrated reproducibility across independent patient cohorts. Finally, we examined transcriptomic and proteomic datasets—including single-cell and spatial transcriptomic analyses—that have collectively uncovered tumor microenvironment heterogeneity, immunosuppressive mechanisms, and novel therapeutic vulnerabilities. Together, these six interrelated domains reflect the progressive shift in chordoma research from purely morphological toward integrated molecular classification, with direct implications for prognostication, therapeutic targeting, and clinical trial design in this rare and treatment-refractory malignancy.

There exists an urgent need to consolidate the various classification approaches into clinically consistent groups that can be acted upon. Some clear patterns emerge from the reviewed articles. The first theme is associated with CNAs. Variable level of CIN have been independently identified in three cohorts of patients [8,59,120], and this score is clearly prognostic [8,59,120]. *CDKN2A/B* loss is associated with a distinct DNA methylation pattern and worse prognosis; however, it is debated if the loss of chr9p is a better prognostic factor [8,10,54,62,82]. The importance of cell cycle in chordoma biology is underpinned by studies showing *CDK9* and *CDK12* expression to be prognostic [110,111]. Beyond *CDKN2A/B* loss, patient stratification based on CNA is linked to the transcriptomic profile and immune cell infiltration [64]. Hopefully, further research will allow establish if there is a place for theranostic use of *CDKN2A/B* status. So far, *CDKN2A/B* loss has been an entry criterion in a single-arm phase II trial of palbociclib (CDK4/6 inhibitor). The antitumor activity was modest (11/59 patients achieved stable disease, and no objective response was identified) [63].

There exists a clear variability in immune microenvironment of chordoma, consistently evident across transcriptomic, epigenomic, and proteomic classification frameworks. Single-cell RNA sequencing studies have characterized a tumor microenvironment marked by abundant M2-like macrophages, exhausted CD8^+^GZMK^+^ T cells, and active TGF-β signaling between macrophages and fibroblasts [99], with recurrent tumors showing further immunosuppressive remodeling characterized by reduced antigen-presenting cell activity, fewer plasma cells, and lower BCR clonotype diversity [104]. PD-L1 expression is predominantly localized to tumor-infiltrating macrophages and lymphocytes rather than chordoma cells themselves [106], and a specific inflammatory cancer-associated fibroblast subset—spatially distant from tumor cells—has been functionally linked to promotion of chordoma progression [103]. Collectively, these findings suggest that immunosuppression in chordoma is orchestrated largely through stromal and myeloid compartments rather than intrinsic tumor cell mechanisms. At the epigenomic level, unsupervised methylation clustering consistently identifies an immune-infiltrated subtype characterized by higher cytotoxic T lymphocyte, B cell, and macrophage infiltration and lower tumor purity [8,81,82], with two studies reporting worse prognosis in this subtype [81,82]. Transcriptomic subtyping of SBCs similarly identified two immune subtypes differing in macrophage and T-cell infiltration, with higher *CD68* and *CD163* expression—markers of M2 macrophage polarization—correlating with shorter progression-free and overall survival [96]. Immunotherapy (with immune checkpoint inhibitors) efficacy in chordoma has been evaluated in a retrospective cohort of seventeen patients, showing promising results (one complete response, three partial responses, and eleven cases of stable disease) [121]. An ongoing one-arm phase II clinical trial (NCT06794645) prospectively measures the efficacy of pembrolizumab and pemetrexed in recurrent chordoma. However, to our knowledge, there is no trial that utilizes immune infiltration in chordoma as an inclusion criterion.

Third theme is the dysfunction of SWI/SNF chromatin remodeling complex. At the histological level, homozygous loss of *SMARCB1* defines PDC and is mechanistically linked with EZH2 activity [34,35,36,37,38,39,40,41]. Meanwhile, a distinct Polycomb-type dedifferentiation pathway driven by homozygous *EED* deletion has been identified in a subset of skull base DCs [33]. Heterozygous deletion of chr22q, which harbors *SMARCB1*, is additionally observed in conventional chordoma and was associated with worse disease-specific and recurrence-free survival in one cohort [10], raising the unresolved question of whether heterozygous loss represents a transition state toward poorly differentiated disease. At the genomic level, somatic mutations in *PBRM1*, *SETD2*, and *ARID1A* are among the most frequent driver events across skull base, spinal, and sacral cohorts [10,54,56,57,58]. Therefore, chromatin remodeling dysfunction is a clearly established feature of some, but not all chordomas. The prognostic effect is clear with homozygous deletion of *SMARCB1* in PDC [34,35,36,37,38,39,40,41]. Its theranostic use has been recently explored—one patient with metastatic PDC experienced sustained response to tazemetostat treatment [41], and patient-derived xenografts with *PBRM1* mutation were sensitive to tazemetostat [122].

The final overarching theme concerns the composition of the tumor stroma. Chordoma exhibits marked variability in its stromal content, and the distinction between stroma-rich and stroma-poor tumors has been independently observed across electron microscopy [43] and MRI-based classifications [98]. However, the prognostic significance of this distinction remains unresolved: Bai et al. reported that CDT (stroma-poor) tumors carried a worse prognosis [43], while Park et al. observed only a statistically insignificant trend toward inferior outcomes in stroma-rich tumors [98]. The stromal compartment of chordoma is dominated by cancer-associated fibroblasts, which constitute its primary cellular component [9,103]; stroma-rich tumors, characterized by upregulated collagen and extracellular matrix gene expression [98], likely reflect a cancer-associated fibroblasts-enriched microenvironment, though this correspondence has not been directly demonstrated. TGF-β is the canonical driver of ECM remodeling across solid tumors, with TGF-β-activated fibroblasts shown to define poor-prognosis stromal subtypes in patient-derived models [123,124]. Consistent with this, TGF-β signaling occurs predominantly between macrophages and fibroblasts within the tumor stroma [99], and a partial epithelial–mesenchymal transition program, driven by TGF-β, was localized to the invasive tumor edge, independently predicting worse progression-free and overall survival in SBCs [100]. This classification theme is the least developed in comparison to the ones described so far and requires further research.

Comparing the identified themes, all have some proposed utility in prognostication. Immune cell infiltration and CIN have the clearest evidence, while for SWI/SNF dysfunction and stromal composition, conflicting reports exist. Although all themes have the potential to be used to identify therapeutic opportunities, no prospective randomized controlled trial has assessed their utility. There is an open question of how these themes link with each other. For example, *CDKN2A/B* deletions have been reported predominantly outside the immune-infiltrated subtype in one cohort [8], though this association is not consistent across studies [82]. Moreover, *CDKN2A/B* deletion and SWI/SNF mutations appear mutually exclusive in at least one study [54]. Chromatin remodeling also seems to be related to gene expression patterns—RNA sequencing identified two molecular subtypes—CC1 tumors enriched for *PBRM1* and *SETD2* mutations and CC2 tumors characterized by Sonic Hedgehog pathway upregulation [97]. Despite these observations, chordoma classification has not yet been refined by the re-analysis of published molecular studies, as has been the case with other rare tumors (e.g., somatotroph pituitary neoplasms [125] or ependymomas [126]). Only speculative reasons for this deficiency can be offered, but barriers in data and metadata sharing are probably a major contributing factor.

A critical caveat that applies across all four cross-cutting themes identified in this review is the marked imbalance in the anatomical representation of studied cohorts. Many large-scale molecular studies—the transcriptomic CC1/CC2 classification [97], the multi-omics proteogenomic landscape [9], the characterization of the immune microenvironment in bulk RNA-seq [96], and 2/3 studies defining the DNA methylation subtypes [8,82]—were conducted exclusively in SBCs. This imbalance is not trivial: site-specific differences in genomic landscape have been documented, including higher frequencies of driver gene mutations, *TBXT* amplifications, deletions of 5p, 5q, and 9p, as well as a later age of onset in sacral versus clival tumors [10,59,75,127]. Furthermore, histopathological studies have demonstrated that SBCs exhibit more abundant chondroid matrix and a diffuse growth pattern, whereas spinal chordomas more commonly show a lobulating, non-chondroid myxoid architecture [128]. Consequently, the four thematic conclusions of this review—while internally consistent—should be interpreted primarily as a framework for SBCs, with uncertain applicability to sacral and spinal disease.

This review has several limitations that must be acknowledged. Although predefined search strategy was used to identify relevant literature, this review is narrative in nature, and the synthesis of included studies remains subjective. The field itself carries limitations that are difficult to mitigate: the rarity of chordoma means that the vast majority of published studies are retrospective in nature, and most cohorts comprise fewer than 100 patients, limiting statistical power and increasing the risk that reported associations will not replicate in independent datasets. Furthermore, the studies were conducted across anatomically distinct disease sites and in heterogeneous patient populations, making cross-cohort comparisons inherently difficult. Inconsistencies in molecular subtype nomenclature, differing platforms for analysis, and variable clinical endpoint definitions compound this challenge. Finally, the rapid pace of discovery in single-cell sequencing, spatial transcriptomics, and liquid biopsy methodologies means that the evidence base continues to evolve quickly, and some conclusions drawn here will require revision as larger datasets mature. Addressing these limitations will require coordinated international efforts to establish biobanks and prospective registries capable of capturing sufficient patient numbers to validate the classification frameworks described in this review and link them rigorously to therapeutic outcomes.

## Figures and Tables

**Table 1:** Inclusion criteria for this study.

Element	Description
Population	Patients with histologically confirmed chordoma or whose lesion could have been systematically misclassified as chordoma
Interest	Proposed classification system or subtyping framework
Context	Clinical, molecular, or imaging setting
Outcome	Described subtypes; association with prognosis, treatment response, or biological behavior
Study design	Original research papers that describe more than one patient

## Data Availability

No new data were created or analyzed in this study.

## Supplementary Materials

The following supporting information can be downloaded at: https://www.mdpi.com/article/10.3390/cells15090750/s1, Table S1: StudyTable.csv; Script S1: review.rmd.

## Abbreviations

The following abbreviations are used in this manuscript:

ADC	apparent diffusion coefficient
BNCT	benign notochordal cell tumor
ChoC	chondroid chordoma
CIN	chromosomal instability
CDT	cell-dense type classic chordoma
CNA	DNA copy number event
DC	dedifferentiated chordoma
DKFZ	Deutsches Krebsforschungszentrum
EMA	epithelial membrane antigen
FLAIR	fluid-attenuated inversion recovery
MRI	magnetic resonance imaging
MRT	matrix-rich type classic chordoma
PDC	poorly differentiated chordoma
RT2	ratio of tumor-to-pons signal intensity on T2 sequences
SBC	skull base chordoma
scRNAseq	single-cell RNA sequencing
WHO	World Health Organization
